# A Temporal Role Of Type I Interferon Signaling in CD8^+^ T Cell Maturation during Acute West Nile Virus Infection

**DOI:** 10.1371/journal.ppat.1002407

**Published:** 2011-12-01

**Authors:** Amelia K. Pinto, Stephane Daffis, James D. Brien, Maria D. Gainey, Wayne M. Yokoyama, Kathleen C. F. Sheehan, Kenneth M. Murphy, Robert D. Schreiber, Michael S. Diamond

**Affiliations:** 1 Department of Medicine, Washington University School of Medicine, St. Louis, Missouri, United States of America; 2 Department of Pathology and Immunology, Washington University School of Medicine, St. Louis, Missouri, United States of America; 3 Department of Molecular Microbiology, Washington University School of Medicine, St. Louis, Missouri, United States of America; Mount Sinai School of Medicine, United States of America

## Abstract

A genetic absence of the common IFN- α/β signaling receptor (IFNAR) in mice is associated with enhanced viral replication and altered adaptive immune responses. However, analysis of IFNAR^-/-^ mice is limited for studying the functions of type I IFN at discrete stages of viral infection. To define the temporal functions of type I IFN signaling in the context of infection by West Nile virus (WNV), we treated mice with MAR1-5A3, a neutralizing, non cell-depleting anti-IFNAR antibody. Inhibition of type I IFN signaling at or before day 2 after infection was associated with markedly enhanced viral burden, whereas treatment at day 4 had substantially less effect on WNV dissemination. While antibody treatment prior to infection resulted in massive expansion of virus-specific CD8^+^ T cells, blockade of type I IFN signaling starting at day 4 induced dysfunctional CD8^+^ T cells with depressed cytokine responses and expression of phenotypic markers suggesting exhaustion. Thus, only the later maturation phase of anti-WNV CD8^+^ T cell development requires type I IFN signaling. WNV infection experiments in *BATF3* ^-/-^ mice, which lack CD8-α dendritic cells and have impaired priming due to inefficient antigen cross-presentation, revealed a similar effect of blocking IFN signaling on CD8^+^ T cell maturation. Collectively, our results suggest that cell non-autonomous type I IFN signaling shapes maturation of antiviral CD8^+^ T cell response at a stage distinct from the initial priming event.

## Author Summary

Although it is well established that type I IFN responses protect against viral infections by inducing expression of antiviral genes and modulators of adaptive immune responses, its function at different stages of viral infections has remained poorly studied. In this paper, we administered a monoclonal antibody that blocks the common type I IFN signaling receptor to mice at different times after WNV infection to dissect the temporal functions of IFN. Administration of the blocking antibody at day -1 resulted in a massive increase in viral replication and the number of WNV-specific -CD8^+^ T cells. In contrast, treatment with a single dose of antibody at day 4 had limited effects on viral dissemination, but instead promoted development of dysfunctional CD8^+^ T cells that produced lower levels of cytokines and expressed proteins implicated in T cell exhaustion. Thus, we show a stage-specific effect of type I IFN in optimal maturation of antiviral CD8^+^ T cell responses. Our study provides new insight as to how and when innate immune signals affect maturation of antiviral CD8^+^ T cells after the initial priming event with viral antigen.

## Introduction

Type I interferons (IFN) comprise a family of cytokines that that were identified originally for their ability to render cells resistant to virus infection [1]. Type I IFN binds to a common IFN-αβ receptor (IFNAR), which initiates a signaling cascade that results in phosphorylation and nuclear translocation of STAT1 and STAT2, and induction of expression of hundreds of interferon-stimulated genes (ISG) [2]. These ISG control viral infections through a diverse range of direct antiviral effector functions [3] and by modulating adaptive immune responses [4].

Type I IFN responses are essential for the controlling infection by West Nile virus (WNV) [5], [6], an encephalitic positive strand RNA virus of the *Flaviviridae* family that has emerged over the past decade as a significant cause of neuroinvasive disease [7]. *IFNAR^-/-^* mice are exquisitely vulnerable to WNV infection, with expanded tissue tropism, uncontrolled viral replication, and rapidly uniform death, with all animals succumbing within four days of infection after inoculation with a single plaque forming unit (PFU) of virus [8].

Apart from its function in controlling viral infection through cell-intrinsic antiviral gene induction, type I IFN has an established role in priming of B and T cell responses (reviewed in [9], [10]). Signaling through IFNAR regulates early innate and adaptive B cell activation in the lymph node and spleen [11]–[13] and induces dendritic cells to mature, express higher levels of co-stimulatory molecules, and present antigen more efficiently, which is required for optimal induction of a functional T cell response (reviewed in [14]). Diminished effector functions of memory CD8^+^ T cells in *IFNAR^-/-^* mice have been described after infection with influenza and vaccinia (VV) viruses [15], [16]. This could be due in part, to defects in cross-priming of CD8^+^ T cells, which is believed to require both virus-induced type I IFN [9], [13], [17] and CD8-α dendritic cells [18].

Although cell-type and tissue-specific conditional deletions of IFNAR have been described [19]–[22], the function of type I IFN at discrete stages of viral infection remains unknown. To define the temporal functions of type I IFN signaling in the context of infection by WNV, we utilized a previously reported blocking anti-IFNAR monoclonal antibody (MAb MAR1-5A3), which prevented type I IFN-induced intracellular signaling in vitro, was non-cell-depleting, and inhibited antiviral, antimicrobial, and antitumor responses in mice [23].

By administering MAR1-5A3 antibody at different times after viral inoculation, we separated the early innate from the later innate-adaptive functions of type I IFN. Treatment prior to WNV infection resulted in massive expansion of virus-specific CD8^+^ T cells by day 9. However, blockade of type I IFN signaling beginning at day 4 after WNV infection was associated with defects in virus-specific effector CD8^+^ T cells at day 9 including depressed IFN-γ and TNF-α responses and changes in phenotypic markers suggesting altered activation status and CD8^+^ T cell exhaustion that is usually seen during chronic viral infection [24]. This phenotype was not due to direct signaling effects through IFNAR on CD8^+^ T cells and was also observed after vaccinia virus (VV) infection under similar experimental conditions. Experiments in *BATF3* ^-/-^ mice, which lack CD8-α dendritic cells and have impaired antigen cross-presentation and CD8^+^ T cell priming capacity, showed a similar effect of temporal blockade of type I IFN signaling on CD8^+^ T cell maturation. Collectively, our results suggest that cell non-autonomous type I IFN signaling shapes maturation of antiviral CD8^+^ T cell response at a stage distinct from the initial priming event.

## Results

### Blocking the type I IFN receptor at different times results in enhanced susceptibility to WNV

Previous studies established a critical requirement for type I IFN in controlling WNV-NY (strain New York, 2000) as infected *IFNAR^-/-^* mice showed expanded tissue tropism, uncontrolled viral replication, and rapidly uniform death within four days [8]. While these experiments suggested a dominant antiviral function of type I IFN in vivo, key roles in modulating adaptive B and T cell responses against viruses also have been described [13], [17]. One caveat of the antiviral and immunologic studies is that they have been performed primarily in complete or cell-type *IFNAR* ^-/-^ mice, which limits insight into the temporal function of IFN signaling in modulating immune responses. Also, because many viruses replicate to substantially higher levels in *IFNAR* ^-/-^ mice, it can be difficult to separate how enhanced antigen burden and lack of type I IFN signaling differentially impact adaptive immune responses in the context of live virus infection. To begin to define the temporal functions of type I IFN signaling, we utilized MAR1-5A3, a previously described MAb that potently blocks type I IFN receptor signaling and is non cell-depleting [23].

**Figure 1:**
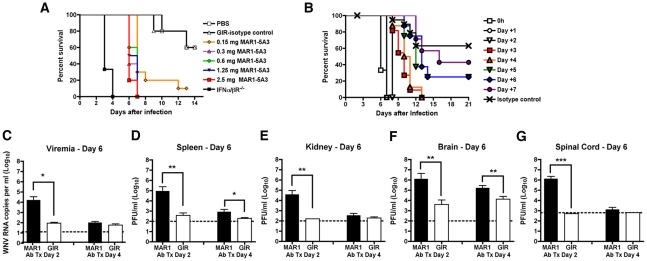
Effect of blockade of type I IFN signaling on WNV-NY infection. **A**. Dose titration of the MAR1-5A3 MAb in mice. Mice (*n* = 10 per group) were treated with increasing doses of MAR1-5A3 MAb, infected one day later with 10^2^ PFU of WNV-NY, and survival was monitored. All MAR1-5A3 treatment doses shown were statistically different compared to GIR-208 treatment (*P*<0.05). **B**. Time course of MAR1-5A3 addition after WNV infection. Mice (*n* = 5 to 10 per group) were infected with 10^2^ PFU of WNV-NY, treated with 1 mg of MAR1-5A3 or an isotype control (GIR-208 (GIR)) at different times after infection, and survival was determined. Treatment with MAR1-5A3 at days 0, 1, 2, 3, and 4 were statistically different (*P*<0.004) compared to treatment with GIR-208. **C–G**. Effect of MAR1-5A3 on viral burden. Mice (*n* = 5 to 10 per group) were infected with 10^2^ PFU of WNV-NY, treated with 1 mg of MAR1-5A3 or GIR at day 2 or day 4 after infection. (**C**) Serum, (**D**) spleen, (**E**) kidney, (**F**) brain, and (**G**) spinal cord were harvested at day six and viral titers were determined by plaque assay or qRT-PCR. Asterisks indicate differences that are statistically significant (*, *P*<0.05; **, *P*<0.01, ***, *P*<0.001).

*IFNAR* ^-/-^ mice succumb to lethal WNV-NY infection within 4 days of infection after a dose of 10^2^ PFU of virus [8]. We assessed whether treatment with the MAR1-5A3 MAb recapitulated this phenotype. We performed a dose titration of MAR1-5A3, in which mice were treated one day prior to infection with 10^2^ PFU of WNV-NY and monitored for survival (**Figure 1A**). Similar to *IFNAR* ^-/-^ mice, all wild type mice treated with a single dose of MAR1-5A3 but not the isotype control GIR-208 MAb ranging from 0.3 to 2.5 mg succumbed to WNV-NY infection, although the mean time of death (MTD) was delayed (6.5 days versus 4 days, *P*<0.0001). Given this data, we chose a MAR1-5A3 dose of 1 mg per mouse for the remainder of the study. The difference in MTD was not unexpected as MAR1-5A3 is not expected to cross the blood-brain-barrier efficiently and type I IFN has direct antiviral effects on neurons in the central nervous system [8], [25], [26].

We hypothesized that type I IFN signaling may have distinct functions at different stages of viral infections. To test this, mice were treated with a single 1 mg dose of MAb at different days after infection and survival was monitored (**Figure 1B**). We observed a significant difference (*P*<0.0003) in survival of mice treated with MAR1-5A3 as late as four days after infection as compared to the isotype control MAb treated mice. The MTD after WNV-NY infection for mice receiving MAR1-5A3 between days 0 and 2 was ∼8 days whereas those receiving MAb on days 3 or 4 survived on average between 10 and 11 days.

To further characterize the impact of type I IFN signaling at different stages, we compared viral titers from organs of mice at day 6 after infection in mice treated with the MAR1-5A3 or the control GIR-208 MAb at days 2 or 4 post infection (**Figure 1C to G**). In mice treated with MAR1-5A3 two days after infection, we observed an increase in viremia (739-fold, *P*<0.02), and infection in the spleen (242-fold, *P*<0.02) and kidney (240-fold, *P*<0.002) compared to the isotype control MAb. This corresponded with markedly higher viral titers in the brain (325-fold, *P*<0.006) and spinal cord (2,650-fold, *P*<0.001) compared to the control group. In contrast, mice treated with a single dose of MAR1-5A3 at day 4 after infection showed substantially smaller increases in the spleen (4.4-fold, *P*<0.03) and brain (13-fold, *P*<0.006) with no detectable elevation in serum, kidney, or spinal cord (*P*>0.19) at day 6. Thus, although the relative timing (day 2 or 4) of MAR1-5A3 administration did not differentially affect clinical outcome, it impacted viral spread and tropism; earlier blockade of type I IFN signaling resulted in enhanced replication in all tissues examined, whereas later administration had a small effect in only a subset of organs.

### Temporal effects of MAR1-5A3 on adaptive immunity against WNV

Several groups have observed differences in antibody and CD8^+^ T cell responses in *IFNAR* ^-/-^ and *STAT1* ^-/-^ mice after infection or vaccination [13], [15]–[17], [19], [27], [28]. Because administration of MAR1-5A3 at day 4 had relatively minor effects on viral burden at day 6 (**Figure 1**) or day 8 (data not shown) yet still resulted in complete lethality, we hypothesized that blockade of type I IFN receptor signaling at later stages might impact early adaptive immune responses.

The development of an antibody response is critical for surviving WNV infection [29], [30]. To study the temporal effects of type I IFN signaling on the humoral response, wild type mice were infected with WNV-NY, treated with MAR1-5A3 or isotype control antibody two or four days later, and serum was harvested at day 6 or 9 after infection. We detected no statistically significant difference in WNV-specific IgM or IgG response between the MAR1-5A3 and control antibody-treated groups at any of the time points tested (**Figure S1**, *P*>0.2). Thus, blockade of type I IFN signaling at day 2 or 4 after infection had no major impact on induction of WNV-specific antibody responses during the acute phase of infection.

**Figure 2:**
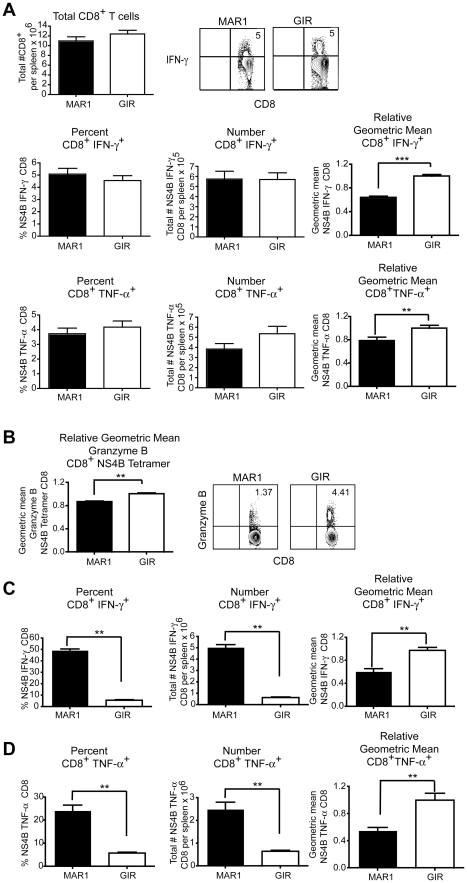
Effect of treatment of MAR1-5A3 on WNV-specific CD8^+^ T cell responses. **A-B**. Mice were infected with 10^2^ PFU of WNV-NY and treated with 1 mg of MAR1-5A3 or GIR-208 (GIR) at day 4 post infection. **A**. Analysis of the CD8^+^ T cell from the spleen of infected MAb-treated mice (*n* = 20 to 25 per group). Splenocytes were harvested on day 9 after WNV infection, and intracellular cytokine staining of IFN-γ and TNF-α was analyzed in CD8^+^ T cells after ex vivo restimulation with NS4B peptide. (*Top left*) Total number of splenic CD8^+^ T cells after infection and treatment with MAbs. (*Top right)* A representative contour plot showing intracellular IFN-γ levels on CD8^+^ T cells after MAb treatment is shown. The percentage, number, and relative staining of WNV-specific IFN-γ^+^ CD8^+^ T cells (*middle panels*) or WNV-specific TNF-α^+^ CD8^+^ T cells (*bottom panels*) are shown. Relative intracellular cytokine staining reflects pooling of data from independent experiments after normalization within a given experiment. **B**. The levels of intracellular granzyme B in WNV-specific CD8^+^ T cells were assessed by co-staining with D^b^-NS4B tetramer and antibodies to granzyme B (*n* = 6 mice per group). A representative contour plot showing intracellular granzyme B levels on CD8^+^ T cells after MAb treatment is shown. **C–D**. Mice were infected with 10^2^ PFU of WNV-MAD and treated with 1 mg of MAR1-5A3 or GIR-208 (GIR) at day -1 and +4 relative to infection. The percentage, number, and relative staining of WNV-specific (**C**) IFN-γ^+^ CD8^+^ T cells or (**D**) TNF-α^+^ CD8^+^ T cells are shown (*n* = 5 mice per group). Asterisks indicate differences that are statistically significant (*, *P*<0.05; **, *P*<0.01, ***, *P*<0.001).

CD8^+^ T cells contribute to the rapid clearance of WNV infection from peripheral and central nervous system (CNS) tissues [31]–[34]. Analysis of CD8^+^ T cells at day 9 in the spleen of wild type mice treated with the MAR1-5A3 or control antibody at day 4 after infection showed a similar percentage and total number of WNV-specific CD8^+^ T cells when measured by intracellular IFN-γ and TNF-α staining after ex vivo incubation with an immunodominant D^b^-restricted NS4B peptide (**Figure 2A**) or direct tetramer staining (data not shown). Nonetheless, blockade of type I IFN signaling at day 4 resulted in a decrease in the amount of intracellular IFN-γ (*P*<0.0001) and TNF-α (*P*<0.006) produced by individual antigen-specific CD8^+^ T cells as judged by differences in the geometric mean fluorescence intensity of the positive cells. Correspondingly, the amount of granzyme B in NS4B tetramer positive cells was less (*P*<0.003) in MAR1-5A3 treated mice (**Figure 2B**). The differences in intracellular cytokines and granzyme B protease establish a late temporal role for type I IFN signaling in the maturation of the antigen-specific CD8^+^ T cells, even though initial priming, as reflected by the total percentage and number of WNV-specific IFN-γ^+^ CD8^+^ or TNF-α^+^ CD8^+^ T cells, remained intact.

To determine whether a similar effect on T cell maturation was observed if type I IFN was neutralized throughout infection, we pre-treated mice with MAR1-5A3 prior to infection with an attenuated lineage 2 WNV strain from Madagascar (WNV-MAD) [35], [36]. We used this less virulent WNV strain because mice treated with MAR1-5A3 and infected with WNV-NY did not survive past day 6 (see **Figure 1**). In comparison, mice treated with MAR1-5A3 before or after infection with attenuated WNV-MAD showed very limited mortality (data not shown). Accordingly, mice were treated with MAR1-5A3 or isotype control mAb one day prior to and four days after infection with WNV-MAD, and CD8^+^ T cells were analyzed at day 9. Notably, depletion of type I IFN signaling throughout the course of infection resulted in a substantial increase in the percentage (6 to 49%, *P*<0.008) and number (*P*<0.008) of WNV-specific IFN-γ^+^ CD8^+^ T cells (**Figure 2C**). Similar results were observed when intracellular TNF-α was measured (**Figure 2D**). The large increase in CD8^+^ T cell priming may be attributed to the greater WNV antigen burden in lymphoid tissues in mice lacking type I IFN signaling [8]. However, and consistent with that observed with MAR1-5A3 treatment at day 4 only with WNV-NY infection, the amounts of intracellular IFN-γ and TNF-α present in WNV-specific CD8^+^ T cells were lower (*P*<0.008) when type I IFN signaling was blocked throughout infection.

Blockade of type I IFN receptor at day 4 also modulated the CD4^+^ T response after WNV-NY infection. The percentage of IFN-γ^+^ or TNF-α^+^ CD4^+^ T cells, as measured after ex vivo stimulation with anti-CD3 antibodies, was decreased (*P*<0.007) in mice receiving MAR1-5A3 compared to the GIR-208 isotype control MAb (**Figure S2)**. While we observed a significant decrease (*P*<0.004) in the total number of TNF-α^+^ producing CD4^+^ T cells in MAR1-5A3 treated mice, this was not observed in IFN-γ^+^ CD4^+^ T cells. Analogous to that seen with WNV-specific CD8^+^ T cells, decreased amounts (*P*<0.01) of IFN-γ and TNF-α were produced by activated CD4^+^ T cells in animals treated with MAR1-5A3.

### Temporal effect of MAR1-5A3 on regulatory T cell induction

Given that a blockade of type I IFN signaling resulted in WNV-specific CD8^+^ T cells that expressed lower levels of intracellular cytokines, we speculated that this could be due to an increase in CD4^+^CD25^+^FoxP3^+^ regulatory T cells. Type I IFN has been reported to alter regulatory T cell activity, which impacts CD8^+^ T cell function [37], [38], and decreased regulatory T cell levels augment WNV-specific CD8^+^ T cell responses [39]. However, we observed no difference in the percentage or numbers of CD4^+^CD25^+^FoxP3^+^ cells at day 9 in the blood (data not shown) or spleen (*P*>0.6), when MAR1-5A3 or control GIR-208 MAb was administered at day 4 after WNV-NY infection (**Figure S3**).

### Temporal effect of MAR1-5A3 on CD8^+^ T cell responses after VV infection

**Figure 3:**
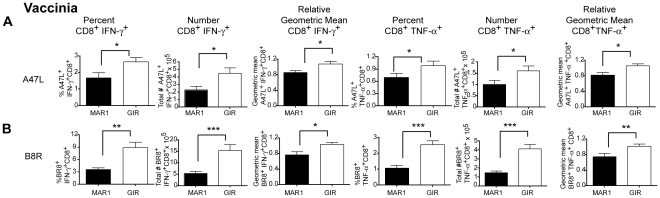
Effect of MAR1-5A3 on VV-specific CD8^+^ T cell responses in the spleen. Mice were infected with 10^4^ PFU of VV (Western reserve strain), treated at day 4 with 1 mg of MAR1-5A3 or GIR-208 MAb, and splenocytes were harvested on at day 9 for intracellular cytokine staining of IFN-γ and TNF-α of CD8^+^ T cells after peptide restimulation with immunodominant A47L (**A**) or B8R (**B**) peptides (*n* = 9 mice per group). Relative intracellular cytokine staining reflects pooling of data from independent experiments after normalization within a given experiment. Asterisks indicate differences that are statistically significant (*, *P*<0.05; **, *P*<0.01, ***, *P*<0.001).

Our data suggested that type I IFN signaling at a later stage modulated WNV-specific T cell responses despite having limited effects on viral replication or initial priming. To determine whether this finding was typical of other viral infections, we repeated MAR1-5A3 treatments at day 4 after infection with an unrelated DNA (VV, Western reserve strain) virus. Mice were harvested eight days after infection (four days after treatment) and T cell populations were analyzed. Similar to that seen with WNV, the amount of intracellular IFN-γ produced by CD8^+^ T cells from the MAR1-5A3 treated mice was lower (*P*<0.02) after re-stimulation ex vivo with two different VV-peptides (A47L or B8R) compared to the isotype control GIR-208 treated mice (**Figure 3A and B**). Notably, and in contrast to WNV infection, we also detected a decrease in the percentage (*P*<0.02) and number (*P*<0.04) of IFN-γ producing VV-specific CD8^+^ T cells, suggesting that for VV infection, type I IFN signaling at day 4 or after also contributed to initial priming. Similar results were observed with TNF-α production with VV-specific CD8^+^ T cells after MAR1-5A3 treatment (**Figure 3A and B** **).** Thus, a temporal blockade of type I IFN signaling impairs antigen-specific CD8^+^ T cell maturation in the context of infection by WNV and VV, two unrelated RNA and DNA viruses.

### Type I IFN receptor signaling, CD8-α dendritic cells, and CD8^+^ T cell responses

**Figure 4:**
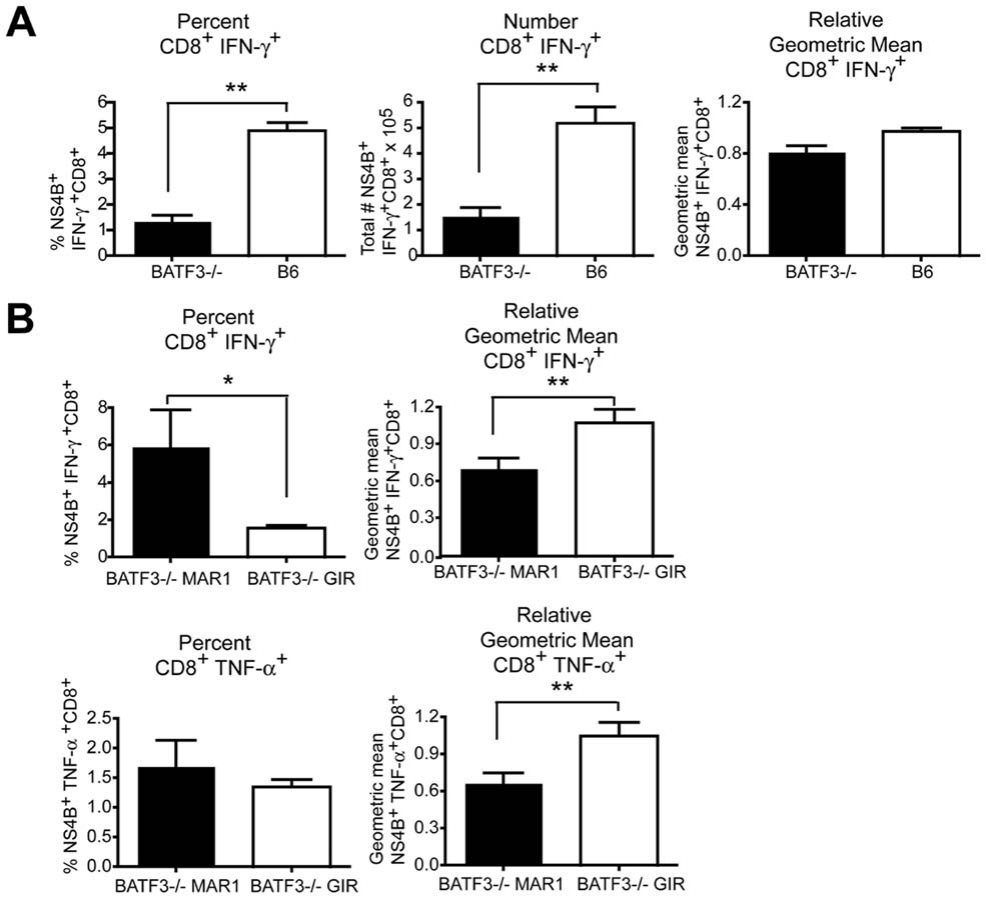
Effect of deletion of BATF3 and loss of CD8-α dendritic cells on CD8^+^ T cell responses after WNV infection. **A**. Wild type or *BATF3* ^-/-^ mice were infected with 10^2^ PFU of WNV-NY, spleens were harvested on day 9 after infection, and intracellular IFN-γ responses in CD8^+^ T cells were measured by flow cytometry after ex vivo stimulation with NS4B peptide (*n* = 5 mice per group). **B**. Wild type or *BATF3* ^-/-^ mice were treated with MAR1-5A3 or GIR-208 (1 mg per dose) at day 4 after infection with 10^2^ PFU of WNV-NY. Spleens were harvested on day 9 after infection, and intracellular IFN-γ (*top panels*) and TNF-α (*bottom panels*) responses in CD8^+^ T cells were measured by flow cytometry after ex vivo stimulation with NS4B peptide (*n* = 5 mice per group). Relative intracellular cytokine staining reflects pooling of data from independent experiments after normalization within a given experiment. Asterisks indicate differences that are statistically significant (*, *P*<0.05; **, *P*<0.01, ***, *P*<0.001).

Recent studies have suggested that type I IFN enhances the CD8^+^ T cell response during antigen cross-presentation [17], [22], [40], [41]. To evaluate whether the temporal effect of type I IFN signaling on CD8^+^ T cell responses occurred in mice with impaired cross-presentation capacity, we utilized *BATF3* ^-/-^ mice, which lack CD8-α and CD103^+^ dendritic cells [18], [42]. Consistent with earlier results from *BATF3* ^-/-^ 129SvEv mice [18], we observed a decrease in the percentage and number (*P*<0.008) WNV-specific CD8^+^ T cells in *BATF3* ^-/-^ mice on the C57BL/6 background although no substantive difference (*P*>0.06) in intracellular IFN-γ levels was detected (**Figure 4A**).

To determine whether mice with priming defects due to impaired cross-presentation still required late stage type I IFN for CD8^+^ T cell maturation, MAR1-5A3 or control GIR-208 MAb was administered to wild type or *BATF3* ^-/-^ mice at day 4 after WNV-NY infection. As expected, associated with the absence of CD8-α dendritic cells, the magnitude (percentage and number) of IFN-γ^+^ and TNF-α^+^ NS4B-specific CD8^+^ T cells at day 9 was markedly lower in MAR1-5A3 or GIR-208 MAb treated *BATF3* ^-/-^ mice compared to wild type animals (data not shown). Nonetheless, reduced intracellular levels of IFN-γ and TNF-α (*P*<0.009) in WNV-specific CD8^+^ T cells were still observed in *BATF3* ^-/-^ mice treated with MAR1-5A3 at day 4 compared to control MAb-treated animals (**Figure 4B**). Thus, the temporal effect of type I IFN blockade on CD8^+^ T cell maturation occurred both in the presence or absence of CD8-α dendritic cells and efficient antigen cross-presentation.

### Cell-extrinsic effect of type I IFN modulates CD8^+^ T cell functional development

Studies with *IFNAR* ^-/-^ bone marrow chimera or conditionally deleted IFNAR on T cells showed reduced cross-presentation of ovalbumin peptides to CD8^+^ T cells, suggesting that direct stimulation of T cells by type I IFN enhances the antigen-specific CD8^+^ T cell response, at least for soluble antigens [22]. Blockade of type I IFN signaling four days after WNV infection results in a dysfunctional antigen-specific CD8 T cell population that nonetheless appeared to undergo a relatively normal priming phase. In comparison, MAR1-5A3 treatment at days -1 and 4 (essentially throughout infection) resulted in a dysfunctional antigen-specific CD8^+^ T cell population, but with a massive increase in the fraction and number of antigen-specific T cells. To establish whether the effect of type I IFN on CD8^+^ T cell functional development was cell-intrinsic in the context of viral infection, we adoptively transferred naïve purified *IFNAR* ^-/-^ (CD45.2) or B6.SJL (CD45.1) CD8^+^ T cells into *RAG1* ^-/-^ recipient mice. Immediately after WNV infection, blood was sampled to confirm transfer of T cell populations in the recipient mice (data not shown). At day nine after infection, spleens were harvested and the CD8^+^ T cell activation profiles analyzed. Notably, we did not detect a significant difference (*P*>0.06) in the intracellular levels of IFN-γ or TNF-α between the *IFNAR* ^-/-^ (CD45.2) and B6.SJL (CD45.1) CD8^+^ T cells donor cells in the *IFNAR* ^+/+^ *RAG1* ^-/-^ mice (**Figure S4**). This result suggests that at least in the context of WNV infection, the effect of type I IFN on the development of a functional CD8^+^ T cell response is largely T cell non-autonomous in nature.

### Effect of MAR1-5A3 on antigen-presenting cells

**Figure 5:**
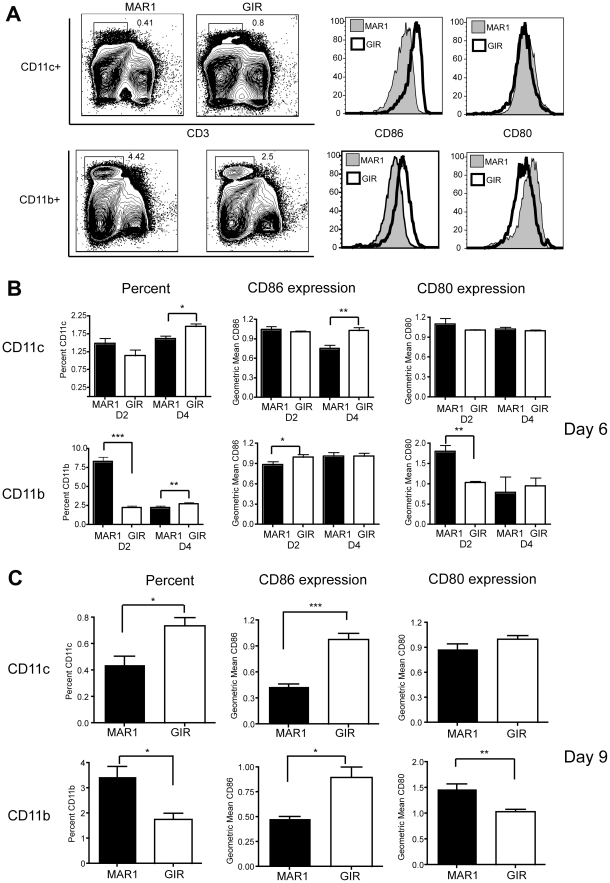
Effect of MAR1-5A3 treatment on costimulatory molecule expression of antigen-presenting cells. Mice were infected with 10^2^ PFU of WNV-NY and treated with 1 mg of MAR1-5A3 or GIR-208 at days 2 or 4 post infection. At days 6 or 9 after infection, CD11b^+^ and CD11c^+^ splenocytes were analyzed for expression of CD80 and CD86 by flow cytometry. **A**. Gating strategy and representative histograms are shown from animals treated with MAR1-5A3 or GIR-208 at day 4 and harvested at day 9. **B-C**. Summary of data showing the percentage of CD11c^+^ and CD11b^+^ splenocytes and the mean fluorescence intensity of CD80 and CD86 staining (*n* = 7 to 9 mice per group) from animals (**B**) treated at days 2 or 4 and harvested at day 6 or (**C**) treated at day 4 and harvested at day 9. Asterisks indicate differences that are statistically significant (*, *P*<0.05; **, *P*<0.01, ***, *P*<0.001).

As our adoptive transfer experiments suggested that efficient WNV-specific CD8^+^ T cell activation did not require cell autonomous type I IFN signaling in CD8^+^ T cells, we assessed whether antigen-presenting cells in the spleen were differentially affected by MAR1-5A3 treatment. MAR1-5A3 was administered 2 or 4 days after WNV infection and APC were examined on days 6 and 9 after infection (**Figure 5A, B, and C**). When MAR1-5A3 was given on day 2 and splenocytes analyzed on day 6, no difference was observed in the percentage of CD11c^+^ cells or their relative expression of the co-stimulatory molecules CD80 and CD86 (**Figure 5B**). We did however, observe an increased percentage of CD11b^+^ splenocytes at this time point, and this was associated with reciprocal decreases and increases in expression of CD80 and CD86, respectively. In comparison, when MAR-5A3 was administered on day 4 after WNV-NY infection and splenocytes analyzed on day 6, we observed a reduced percentage of CD11b^+^ (*P*<0.01) and CD11c^+^ (*P*<0.02) cells, and this was associated with decreased expression of CD86 only on CD11c^+^ cells (**Figure 5B, ***P*<0.008). When MAR-5A3 was administered on day 4 after WNV-NY infection and splenocytes analyzed on day 9, we also observed a decrease in surface expression of CD86 on CD11b^+^ (*P*<0.05) and CD11c^+^ (*P*<0.007) cells relative to the control MAb treatment (**Figure 5A and C**). In comparison, MAR1-5A3 treatment had no effect on CD80 expression on CD11c^+^ cells although an increase (*P*<0.005) was noted in CD11b^+^ cells at this time. Thus, blockade of type I IFN signaling at day 4 after infection resulted in a distinct antigen-presenting cell activation phenotype compared to MAR1-5A3 treatment at day 2; this suggests that disruption of type I IFN signaling pathways at particular stages of infection might limit the ability of antigen-presenting cells to provide key temporal signals that allow optimal generation of antigen-specific effector CD8^+^ T cells.

### Effect of MAR1-5A3 on cytokine levels

**Figure 6:**
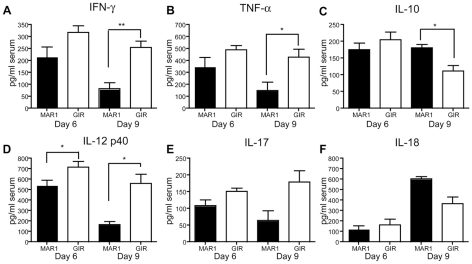
Effect of MAR1-5A3 treatment on serum inflammatory cytokines. Mice were infected with 10^2^ PFU of WNV and treated with 1 mg of MAR1-5A3 or GIR-208 at day 4 post infection. Serum was harvested at day 6 or 9 after infection and analyzed for the (**A**) IFN-γ, (**B**) TNF-α, (**C**) IL-10, (**D**) IL-12 p40, (**E**) IL-17, and (**F**) IL-18 using a Bio-Plex pro cytokine assay (*n* = 6 mice per group). Asterisks indicate differences that are statistically significant (*, *P*<0.05; **, *P*<0.01, ***, *P*<0.001).

We speculated that a specific absence of type I IFN signaling in amtigen-presenting cells impaired development of a WNV-specific CD8^+^ T cell response because of an altered production of cytokines required for maturation. To assess this, we measured the cytokine levels in mice that were treated with MAR1-5A3 at day 4 after WNV-NY infection. Two or five days after MAb treatment (day 6 or 9 after infection), serum was harvested and levels of relevant cytokines (IFN-γ, TNF-α, IL-10, IL-12 p40, IL-17, and IL-18) were measured by bioplex assay (**Figure 6A–F**). Two days after MAR1-5A3 treatment, significantly (*P*<0.04) reduced levels of IL-12 p40 were observed (**Figure 6D** **)**. Within five days of MAR1-5A3 treatment, serum levels of IFN-γ, TNF-α, and IL-12 p40 were reduced significantly (*P*<0.01) and IL-10 was elevated (*P*<0.02). The increased level of IL-10 in mice treated with the blocking type I IFN MAb may be particularly relevant as IL-10 negatively impacts CD8^+^ T cell activation and function [37], [38].

### Phenotype of CD8^+^ T cells in MAR1-5A3 treated mice

**Figure 7:**
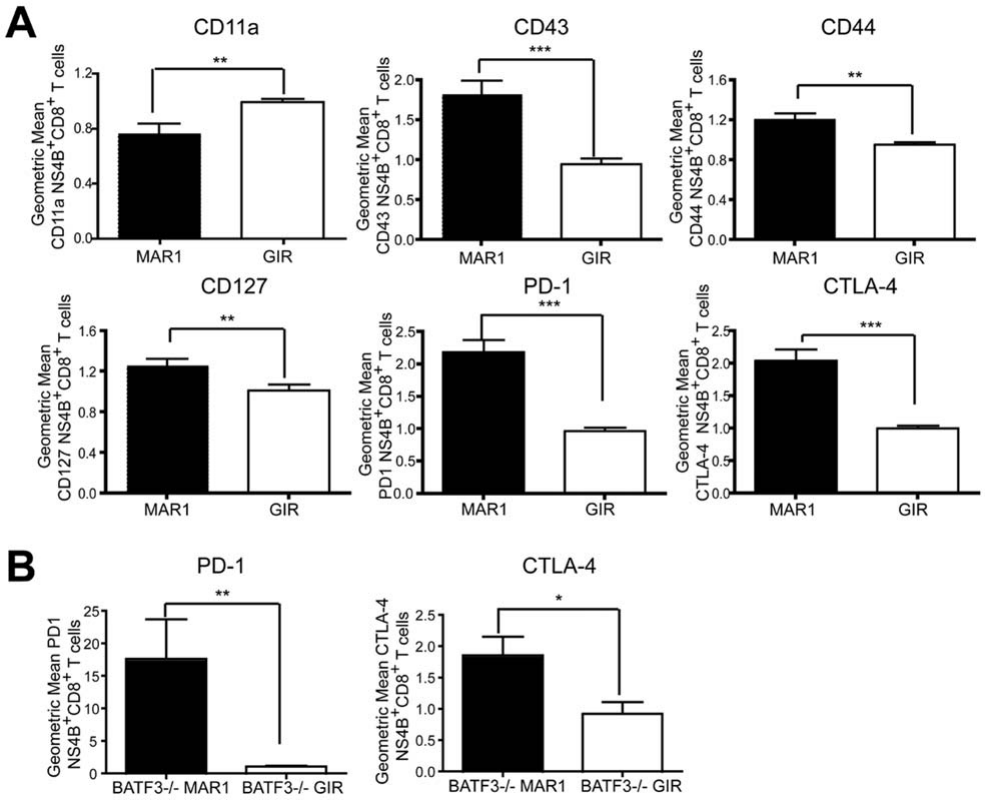
Effect of MAR1-5A3 treatment on expression of markers of CD8^+^ T cell activation and exhaustion. **A**. Wild type mice were infected with 10^2^ PFU of WNV-NY and treated with 1 mg of MAR1-5A3 or GIR-208 at day 4 post infection. Splenocytes were harvested on day 9, co-stained for CD8β and D^b^-NS4B tetramer, and the gated cells analyzed by flow cytometry for relative expression of PD-1, CTLA-4, CD44 CD127, CD11a and CD43 (*n* = 18 mice per group). **B**. *BATF3* ^-/-^ mice were treated with MAR1-5A3 or GIR-208 at day 4 after infection with 10^2^ PFU of WNV-NY. Spleens were harvested on day 9 after infection, co-stained for CD8β and D^b^-NS4B tetramer, and the gated cells analyzed by flow cytometry for relative expression of PD-1 and CTLA-4 (*n* = 5 mice per group). Relative staining reflects pooling of data from independent experiments after normalization within a given experiment. Asterisks indicate differences that are statistically significant (*, *P*<0.05; **, *P*<0.01, ***, *P*<0.001).

Because blockade of IL-10 in chronic lymphocytic choriomengitis virus (LCMV) infection prevents functional exhaustion of CD8^+^ T cells and promotes viral clearance [43], [44], we hypothesized that the increased IL-10 levels in serum of MAR1-5A3 treated mice after WNV-NY infection might cause the CD8^+^ T cells to acquire an exhausted phenotype. To assess this, at day 5 after MAb treatment (day 9 after infection), we profiled D^b^-NS4B-tetramer^+^ CD8^+^ T cells for expression of PD-1, CTLA-4, CD43, CD44, CD127, and CD11a (**Figure 7A**). Notably, treatment with MAR1-5A3 compared to the control MAb resulted in reduced expression of CD11a (*P*<0.001) and increased expression of CD127, CD43, CD44, CTLA-4 and PD-1 (*P*<0.007). Thus, CD8^+^ T cells from mice treated at day 4 with MAR1-5A3 not only showed altered intracellular cytokine patterns (see **Figure 2**) but also displayed some of the phenotypic hallmarks of exhaustion. Similarly, *BATF3* ^-/-^ mice treated with MAR1-5A3 at day 4 after WNV infection also expressed elevated (*P*<0.02) levels of the exhaustion markers CTLA-4 and PD-1 on WNV-specific CD8^+^ T cells at day 9 compared to control MAb (**Figure 7B**). Whereas prior studies described CD8^+^ T cell exhaustion at later time points during chronic LCMV infection [24], [45], blockade of type I IFN signaling independent of the mode of priming appears to exhaust WNV-specific CD8^+^ T cells during the acute effector phase.

One of the earliest stages of CD8^+^ T cell exhaustion is characterized by a reduced capacity to lyse target cells [45], [46]. Although we observed reduced levels of granzyme B in D^b^-NS4B tetramer positive CD8^+^ T cells (**Figure 2**), we questioned whether WNV-specific effector cells during the acute immune response displayed a fully exhausted phenotype. We assessed how MAR1-5A3 treatment affected CD8^+^ T cells ability to lyse peptide pulsed targets in vivo (**Figure S5**). Splenocytes from naïve B6.SJL (CD45.1) mice were divided into two groups: one group was pulsed with NS4B immunodominant peptide and labeled with 500 nM carboxyfluorescein diacetate succinimidyl ester (CFDA), and the other was not pulsed with peptide and labeled with 5 nM CFDA. The two groups were mixed in equal numbers and injected into WNV-infected C57BL/6 (CD45.2) mice at day 9 that had undergone treatment with either MAR1-5A3 or control GIR-208 MAb at day 4. Six hours after labeled cells were transferred, splenocytes were harvested and in vivo killing was assessed. Notably, we observed no difference in killing between the MAR1-5A3 and the control MAb-treated mice (*P*>0.3). Thus, type I IFN blockade at a later stage of WNV infection produces an intermediate exhaustion phenotype with skewed cytokine production, surface expression of exhaustion markers, yet relatively intact cytolytic potential.

## Discussion

In this study, we evaluated the antiviral and immunomodulatory roles of type I IFN signaling after viral infection. While past studies in *IFNAR^-/-^* mice with virulent or attenuated WNV strains revealed enhanced susceptibility, dissemination, and lethality compared to congenic wild type mice [8], [36], [47], they did not address the temporal functions of type I IFN during infection. While administration of MAR1-5A3 at day 2 after infection resulted in markedly enhanced viral burden in multiple tissues as seen in *IFNAR^-/-^* mice [8], treatment at day 4 had more subtle effects on viral replication. Instead, detailed analysis established a key role for later type I IFN signaling in the maturation of effector CD8^+^ T cells. Blockade of type I IFN signaling at day 4 after infection with WNV resulted in depressed cytokine responses and changes in phenotypic markers suggesting altered activation and exhaustion.

Prior studies have reported that type I IFN signaling primes adaptive immune functions including cross-presentation of CD8^+^ T cells, enhancement of antibody responses, and maintenance of dendritic cells in a state competent for antigen-presentation [9], [13], [17], [48]. Depending on the experimental system, type I IFN can act directly on CD8^+^ T cells or indirectly on antigen-presenting cells to influence the fate of CD8^+^ T cells during the initial phases of antigen recognition (reviewed in [49]). Many of these studies used *IFNAR^-/-^* mice [50], adoptive transfer of wild type or *IFNAR^-/-^* immune cells into *IFNAR^-/-^* or wild type mice [27], or cell-type specific deletion of IFNAR [51]. While they have provided significant insight into the immunomodulatory effects of type I IFN and defined key cells involved in priming, they have not elucidated the stage-specific effects of type I IFN. In our experiments, when type I IFN signaling was blocked with MAR1-5A3 prior to infection with an attenuated WNV strain, we observed at day 9 paradoxically enhanced numbers of antigen-specific effector CD8^+^ T cell responses that had deficits in IFN-γ or TNF-α production, results that are consistent with prior infection experiments [52]. The increased numbers of WNV-specific CD8^+^ T cells in mice treated with MAR1-5A3 at day -1 could be due to increased antigen burden or a failure to produce IL-10 and negatively regulate T cell expansion [37].

Administration of a single dose of MAR1-5A3 at day 4 after infection with virulent or attenuated WNV strains revealed a distinct phenotype. Although the absolute percentage and number of NS4B-specific CD8^+^ T cells was similar compared to isotype MAb-treated or unmanipulated animals, the geometric mean fluorescence intensity of IFN-γ or TNF-α was consistently lower. Thus, in the context of WNV infection, the initial priming phase of virus-specific CD8^+^ T cells does not absolutely require type I IFN signaling whereas the later maturation phase does. In addition, MAR1-5A3 treatment on day 4 was associated with lower granzyme B expression, decreased surface levels of the adhesion molecule CD11a (LFA-1), and increased expression of CD44, CD127 (IL-7R α-chain), and CD43 on WNV-specific CD8^+^ T cells. These markers are significant because in mice activated, lytic CD8^+^ T cells display a CD44^hi^ CD43^hi^ CD127^lo^ granzyme B^hi^ phenotype whereas memory CD8^+^ T cells express a CD44^hi^ CD43^lo/int^ CD127^hi^ granzyme B^lo^ phenotype [53]-[55]. Thus, stage-specific blockade of type I IFN signaling alters intracellular cytokine production of antigen-specific CD8^+^ T cells and promotes a transitional phenotype during the acute (day 9) phase that appears to fall somewhere between effector and memory populations.

Consistent with functionally dysregulated CD8^+^ T cells when type I IFN signaling was blocked at day 4, we observed increased expression of PD-1 and CTLA-4, two markers of T cell exhaustion [24], [56], which were originally described in the context of chronic, persistent infection of LCMV [46]. In chronic LCMV infection, there is a hierarchy to CD8^+^ T cell exhaustion with some functions exhausted early (IL-2 production, cytotoxicity, and proliferation) and others persisting longer (intracellular pro-inflammatory cytokines) [45]. In comparison, blockade of type I IFN signaling at day 4 resulted in WNV-specific CD8^+^ T cells at day 9 that retained the ability to kill targets in vivo although they expressed lower quantities of IFN-γ and TNF-α. Thus, stage-specific blockade of type I IFN results in dysfunctional CD8^+^ T cells with loss of some but not all effector functions during the acute phase. Although we cannot address what happens during later stages (evolution and maintenance of memory CD8^+^ T cells) in the context of type I IFN blockade and virulent WNV-NY infection because of complete lethality in the model, kinetic studies are planned with the attenuated WNV-MAD strain and MAR1-5A3 to determine how and when type I IFN signaling affects the transition to and establishment of memory phenotypes.

The dysfunctional CD8^+^ T cell phenotype observed after MAR1-5A3 treatment and WNV infection also was observed after VV infection. The change in CD8^+^ T cell profile with type I IFN blockade at day 4 was even more marked after VV infection, as the percentage, number, and mean fluorescence intensity of antigen-specific CD8^+^ T cells were all significantly reduced at day 9 for two independent immunodominant epitopes. Thus, for VV, late stage type I IFN blockade affected both priming and subsequent maturation.

Cross-priming of CD8^+^ T cells occurs after dendritic cells pick up soluble molecules or cellular debris [57] and are licensed by additional cellular or inflammatory signals [58]. Although type I IFN can license dendritic cells for cross-priming of CD8^+^ T cells with soluble ovalbumin [17], it remains unknown if it is essential in the context of the inflammatory milieu associated with viral infection. We speculated that stage-specific blockade of type I IFN signaling might have dominant effects on CD8^+^ T cells maturation if CD8-α dendritic cells and cross-presentation were required for priming and activation. To evaluate this, we infected *BATF3^-/-^* mice, which lack CD8-α dendritic cells, are defective in antigen cross-presentation, and fail to optimally prime CD8^+^ T cell responses [18]. While the percentage and number of WNV-specific IFN-γ^+^ CD8^+^ T cells was blunted in *BATF3^-/-^* mice, the remaining CD8^+^ T cells that were presumably primed by a distinct antigen presentation pathway showed reduced intracellular cytokine levels and enhanced expression of CTLA-4 and PD-1. Thus, at least during WNV infection, the temporal effects of type I IFN signaling on effector CD8^+^ T cell maturation occur regardless of the initial priming pathway.

Although prior studies have suggested that direct stimulation of T cells by type I IFN enhances ovalbumin-specific CD8^+^ T cell responses during cross-priming [22], we did not observe this in the context of WNV infection. CD45.2 CD8^+^ T cells lacking IFNAR showed roughly equivalent WNV-specific responses compared to congenic CD45.1 CD8^+^ T cells after transfer into and infection of *RAG1* ^-/-^ recipient mice. An analogous small impact of direct stimulation by type I IFN on CD8^+^ T cells was observed after infection with VV [59] but not with LCMV [27], [50]. The differential requirement for direct signaling on CD8^+^ T cells may be due to differences in local and systemic type I IFN production during infection with different pathogens [50].

Blockade of type I IFN at day 4 after WNV infection was associated with decreased expression of CD86 on antigen-presenting cells, which likely influences optimal antigen presentation to CD8^+^ T cells [14], [60]. Indeed, lower levels of pro-inflammatory dendritic cell-produced cytokines (IL-12) [61] that regulate CD8^+^ T cell expansion and activation state were observed in mice treated with MAR1-5A3 at day 4. Alternatively, blockade of type I IFN signaling at day 4 could affect CD8^+^ T cell activation because of the elevated levels of the inhibitory cytokine IL-10. Although our results point to a critical temporal role of type I IFN signaling in the functional activation of CD8^+^ T cells in the context of infection by WNV, future studies are required to define the precise spatial and cell-type specific cues that govern this process.

The administration of a neutralizing anti-IFNAR antibody at day 2 after infection limited the ability of the host to control WNV replication and spread to target tissues, thus confirming a dominant antiviral effect of type I IFN during the early stages of pathogenesis. In comparison, administration of the anti-IFNAR antibody at day 4 after WNV infection had marginal effects on viral replication, no effect on the magnitude of CD8^+^ T cell priming, yet profoundly impacted the functional CD8^+^ T cell responses during the acute effector phase, resulting in blunted cytokine production, and changes in phenotypic markers associated with altered activation status and CD8^+^ T cell exhaustion. Given that several studies have established a protective clearance role for CD8^+^ T cells in the brain after WNV infection with virulent North American strains [31], [33], [34], [62], it is not surprising that a temporally defective type I IFN response that affects optimal CD8^+^ T cell maturation resulted in enhanced lethality.

Future studies that administer neutralizing antibodies against IFNAR, other individual IFN subtypes, or other anti- or pro-inflammatory cytokines at different phases of acute virus infection may reveal stage-specific requirements for shaping effector CD8^+^ T cells, the contraction phase, and the transition to central and effector memory. Such studies, coupled with experiments in mice with cell-type specific deletions of IFNAR, will provide new insight into the spatial-temporal dynamics of CD8^+^ T cell expansion and development during infection by different viruses.

## Materials and Methods

### Ethics statement

This study was carried out in strict accordance with the recommendations in the Guide for the Care and Use of Laboratory Animals of the National Institutes of Health. The protocol was approved by the Institutional Animal Care and Use Committee at the Washington University School of Medicine (Assurance Number: A3381-01). All inoculation and experimental manipulation was performed under anesthesia that was induced and maintained with ketamine hydrochloride and xylazine, and all efforts were made to minimize suffering.

### Viruses and cells

The lineage 1 WNV strain 3000.0259 (WNV-NY) was isolated in New York in 2000 [63] and passaged twice in C6/36 *Aedes albopictus* cells. The lineage 2 WNV strain from Madagascar (DakAnMg798, WNV-MAD) was isolated in 1978 and passaged on C6/36 cells [35]. BHK21-15 cells were used for plaque assay experiments with WNV. VV (Western Reserve) was grown in Vero cells and purified by ultracentrifugation through a 36% sucrose cushion.

### Mice

Wild type and *RAG1* ^-/-^ C57BL/6 mice were obtained commercially (Jackson Laboratories). C57BL/6.SJL-Ptprc^a^/BoyAiTac (B6.SJL, CD45.1) mice were purchased (Taconic). *IFNAR* ^-/-^ mice were obtained from J. Sprent (Scripps Institute, San Diego CA) and were backcrossed ten times onto the C57BL/6 background. *BATF3* ^-/-^ mice [18] were backcrossed onto a C57BL/6 background for ten generations. All mice were housed in the pathogen-free mouse facility at the Washington University School of Medicine. Mice (8 to 12 week-old) were inoculated subcutaneously via footpad injection with 10^2^ plaque-forming units (PFU) of WNV-NY or WNV-MAD. VV (10^4^ PFU) was injected via an intraperitoneal route. MAR1-5A3 (mouse anti-mouse IFNAR, IgG1) or isotype control GIR-208 (mouse anti-human IFN-γ receptor 1, IgG1) MAbs [23] were administered as a single dose at 1 mg per mouse unless otherwise indicated by intraperitoneal (IP) injection at specific times with respect to viral infection. MAR-5A3 and GIR-208 MAbs were purchased (Leinco Technologies) and certified as free of endotoxin contamination and aggregates. The half-life of MAR1-5A3 is reported as 5.2 days when a sufficient amount is administered to saturate the receptor pool [23].

### Quantification of viral burden

For analysis of viral burden MAR1-5A3 or GIR-208 was administered two or four days after infection, and organs were recovered on day 6 after cardiac perfusion with 10 ml of PBS. Tissues were weighed, homogenized using a bead-beater apparatus, and titrated for WNV by plaque assay on BHK21-15 cells as described previously [29]. Serum was obtained from whole blood after phlebotomy of the axillary vein immediately before sacrifice and viremia was measured by analyzing WNV RNA levels using fluorogenic quantitative RT-PCR (qRT-PCR) as described [25].

### WNV-specific antibody analysis

WNV-specific IgM and IgG levels were determined using an envelope (E) protein–specific ELISA as described [64].

### CD4^+^ and CD8^+^ T cell analysis

Intracellular staining of TNF-α and IFN-γ from splenocytes was performed as described previously [33]. Briefly, spleens were harvested and homogenized to form a single cell suspension. Cells (2×10^6^ cells) were added to a 96 well plate and incubated with 2 µg/ml brefeldin A (Sigma) for 6 h at 37°C with 10^−6^ M of immunodominant T cell peptides (WNV: D^b^-restricted NS4B 2488–2496 (SSVWNATTA) [33]; and VV: K^b^-restricted A47L 138–146 (AAFEFINSL) and B8R 20–27 (TSYKFESV) [65]) or 2 µg/ml anti-CD3 (145-2C11) (BD Biosciences). After incubation, the cells were stained with directly labeled antibodies (all from BD Biosciences unless indicated) against CD4 (GK1.5), CD19 (6D5), CD43 (1B11), CD127 (SB/199), CD8β (YTS156.7.7), CD44 (MI7), PD-1 (RMP1-30), and CTLA-4 (UC10-4B9, Biolegend). D^b^-NS4B tetramer was obtained from the NIH tetramer core facility. Cells were washed, fixed, and permeabilized with FixPerm Buffer (eBioscience), and stained intracellularly for anti-IFN-γ (XMG1.2), anti-TNF-α (MP6-XT22, eBioscience), or anti-granzyme B (GB12, Invitrogen). Lymphocytes were processed on an LSRII (BD Bioscience) using FACSDiva 6.1.1 software (BD Bioscience) and analyzed with FlowJo (Treestar). The total numbers of IFN-γ or TNF-α expressing CD4^+^ or CD8^+^ T cells was determined by multiplying the percentage of IFN-γ^+^ or TNF-α^+^ CD4^+^ or CD8^+^ T cells by the total numbers of splenocytes. CD4^+^CD25^+^FoxP3^+^ regulatory T cells were measured using a specific staining kit (eBioscience) following manufacturer's protocol.

#### Adoptive transfer experiments

Splenocytes from naïve wild type (CD45.1) or *IFNAR* ^-/-^ (CD45.2) mice were harvested. CD8^+^ T cells were isolated by negative selection after mixing splenocytes with biotinylated antibodies specific for CD4, NK1.1, B220, and MHC class II (eBioscience). After incubation with anti-biotin beads (Miltenyi Biotec), CD8^+^ T cells were collected (∼85 percent purity) in the flow-through fraction. Wild type or *IFNAR* ^-/-^ CD8^+^ T cells (3×10^6^) were transferred into *RAG1* ^-/-^ recipient mice. One day later, mice were inoculated with 10^2^ PFU of WNV, and nine days post-infection, splenocytes were harvested and analyzed by flow cytometry as described above.

### Cytokine bioplex assay

The cytokine bioplex assay was performed on serum samples from mice at day 6 and day 9 post-infection from WNV-infected mice that had received either MAR1-5A3 or GIR-208 (1 mg/mouse) at day 4 after infection. The BioPlex Pro Assay was performed according to the manufacturer's protocol (BioRad). The cytokine screen included IL-2, IL-4, IL-10, IL-12p40, IL-12p70, IL-15, IL-17, IL-18, IFN-γ, and TNF-α.

### In vivo cytolysis assay

In vivo killing of target cells was performed as previously described [66]. Briefly, splenocytes from B6.SJL (CD45.1) mice were isolated. Half of the cells were labeled with carboxyfluorescein diacetate succinimidyl ester (CFDA) at 500 nM and the remainder was labeled with 5 nM CFDA. After labeling, cells labeled with 500 nM CFDA were pulsed for one hour at 37°C with 1 µM NS4B 2488–2496 peptide, whereas the 5 nM CFDA cells were not pulsed with peptide. Both sets of cells were counted and equal numbers were mixed and injected intravenously (10^7^ cells total per mouse) into recipient WNV-infected (at day 9 after infection) or naïve mice that had received either MAR1-5A3 or GIR-208 (1 mg/mouse) at day 4 post-infection. After 8 hours, the mice were sacrificed and splenocytes were gated on CD45.1 cells (donor cells). The percent killing of target cells was calculated: (1 – (ratio immune/ratio naive)) x 100. Ratio equals the number of NS4B peptide-coated targets/number of reference targets [67].

### Statistical analysis

For survival analysis, Kaplan-Meier curves were analyzed by the log rank test. Statistical significance of viral burden, antiviral antibody titers, and number of activated T cells were analyzed by the Mann-Whitney test. All statistical analysis was performed using Prism software (GraphPad Prism).

## Supporting Information

Figure S1 **Effect of MAR1-5A3 treatment on WNV-specific B cell responses.** Mice were infected with 10^2^ PFU of WNV and treated with 1 mg of MAR1-5A3 or GIR-208 at day 2 or 4 post infection (*n* = 5 to 9 mice per group). Serum was harvested at day 6 or 9 after infection and analyzed for WNV-specific IgM and IgG reactivity by ELISA using recombinant E protein. The differences in antibody levels were not statistically significant. WNV-specific IgG was not analyzed at day 6, as titers are not evident until day 7 after infection.
(TIF)

Figure S2 **Effect of day 4 treatment of MAR1-5A3 on WNV-specific CD4^+^ T cell responses.** Mice were infected with 10^2^ PFU of WNV and treated with 1 mg of MAR1-5A3 or GIR at day 4 post infection (*n* = 23 mice per group). Intracellular IFN-γ (*top*, histograms; and *middle*, data summary) and TNF-α (*bottom*, data summary) responses were measured after ex vivo stimulation with anti-CD3 MAb. Asterisks indicate differences that are statistically significant (*, *P*<0.05; **, *P*<0.01, ***, *P*<0.001).
(TIF)

Figure S3 **Effect of MAR1-5A3 on Treg development in WNV-infected mice.** **A**. Mice were infected with 10^2^ PFU of WNV and treated with 1 mg of MAR1-5A3 or GIR-208 at four days post infection (*n* = 8 to 9 mice per group). At day nine after infection, the percentage and number of Tregs from the spleen was determined after staining for CD4, CD25, and FoxP3 and flow cytometric analysis.
(TIF)

Figure S4 **Cell-extrinsic effect of type I IFN signaling in CD8^+^ T cells on IFN-γ and TNF-α production.** An equal number (10^6^ cells) of naïve CD45.2 *IFNαβR* ^-/-^ or CD45.1 B6.SJL purified CD8^+^ T were adoptively transferred into *RAG1* ^-/-^ recipient mice (*n* = 5 mice per group). The following day the mice were infected with 10^2^ PFU of WNV and also phlebotomized to confirm CD8^+^ T cell transfer (data not shown). At day nine, spleens were harvested and intracellular IFN-γ responses in CD45.1 and C45.2 CD8^+^ T cells were measured by flow cytometry after ex vivo stimulation with the D^b^-restricted NS4B peptide. (*Top panel*) Adoptive transfer strategy. (*Middle panels*) Gating strategy to distinguish cells from different donors in recipient mice. (*Bottom panels*) Percentage and relative mean fluorescence intensity of IFN-γ^+^ and TNF-α^+^ CD8^+^ T cells.
(TIF)

Figure S5 **Effect of MAR1-5A3 treatment on CD8^+^ T cell cytotoxicity in vivo.** At day 9 after infection, CFDA-labeled peptide-pulsed CD45.1 naive target cells (splenocytes from B6.SJL mice) were transferred to MAR1-5A3 or GIR-208-treated WNV-infected or naïve mice (*n* = 6 per group), and six hours later, mice were sacrificed and splenocytes analyzed for the ratio of peptide pulsed to non-pulsed cells by interrogating cells in the CD45.1 gate. The percentage of target cell killing in vivo was calculated by determining the ratio of peptide-pulsed versus unpulsed cells for each mouse, and by normalizing to that seen in naive mice.
(TIF)
