# Supplementary figures and images for: A Temporal Role Of Type I Interferon Signaling in CD8+ T Cell Maturation during Acute West Nile Virus Infection
Source: PLoS Pathog. 2011 Dec 1;7(12):e1002407. doi: 10.1371/journal.ppat.1002407 (PMC3228803; doi:10.1371/journal.ppat.1002407)

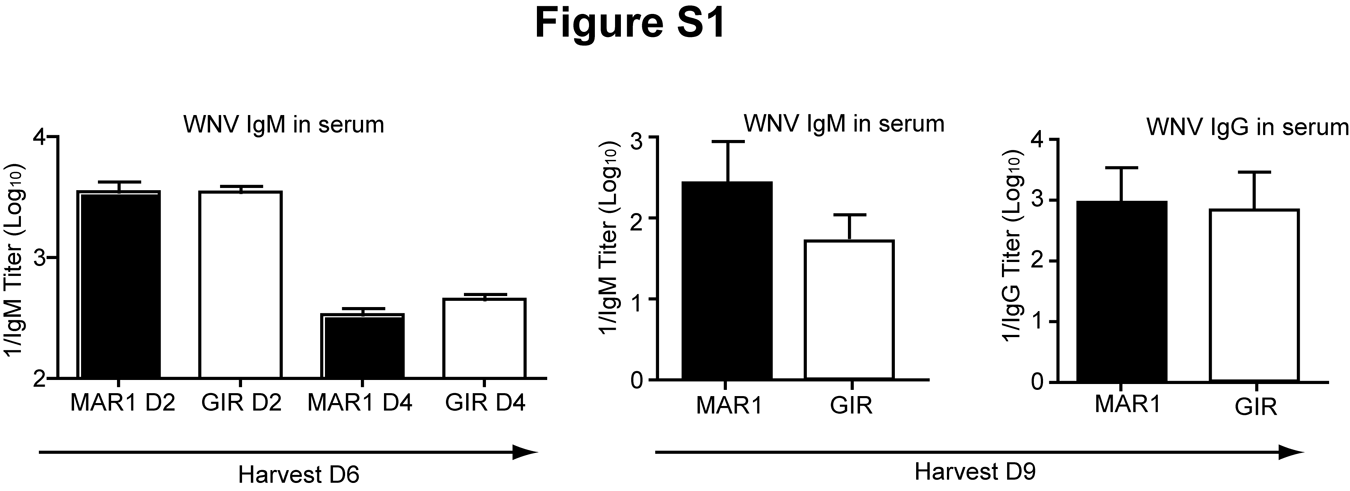

Supplement: Figure S1 — Effect of MAR1-5A3 treatment on WNV-specific B cell responses. Mice were infected with 102 PFU of WNV and treated with 1 mg of MAR1-5A3 or GIR-208 at day 2 or 4 post infection (n = 5 to 9 mice per group). Serum was harvested at day 6 or 9 after infection and analyzed for WNV-specific IgM and IgG reactivity by ELISA using recombinant E protein. The differences in antibody levels were not statistically significant. WNV-specific IgG was not analyzed at day 6, as titers are not evident until day 7 after infection. (TIF) [file ppat.1002407.s001.tif]

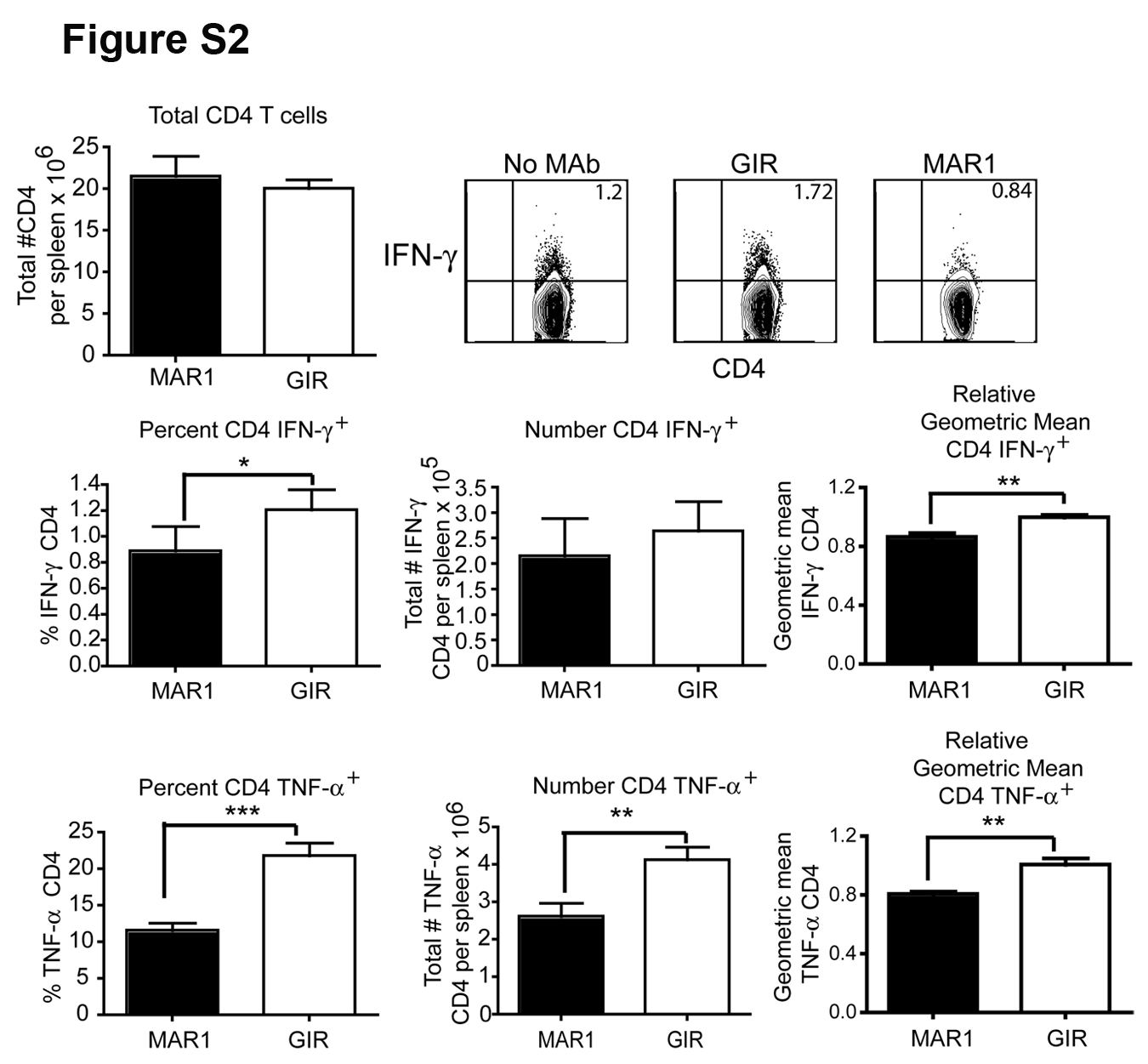

Supplement: Figure S2 — Effect of day 4 treatment of MAR1-5A3 on WNV-specific CD4+ T cell responses. Mice were infected with 102 PFU of WNV and treated with 1 mg of MAR1-5A3 or GIR at day 4 post infection (n = 23 mice per group). Intracellular IFN-γ (top, histograms; and middle, data summary) and TNF-α (bottom, data summary) responses were measured after ex vivo stimulation with anti-CD3 MAb. Asterisks indicate differences that are statistically significant (*, P<0.05; **, P<0.01, ***, P<0.001). (TIF) [file ppat.1002407.s002.tif]

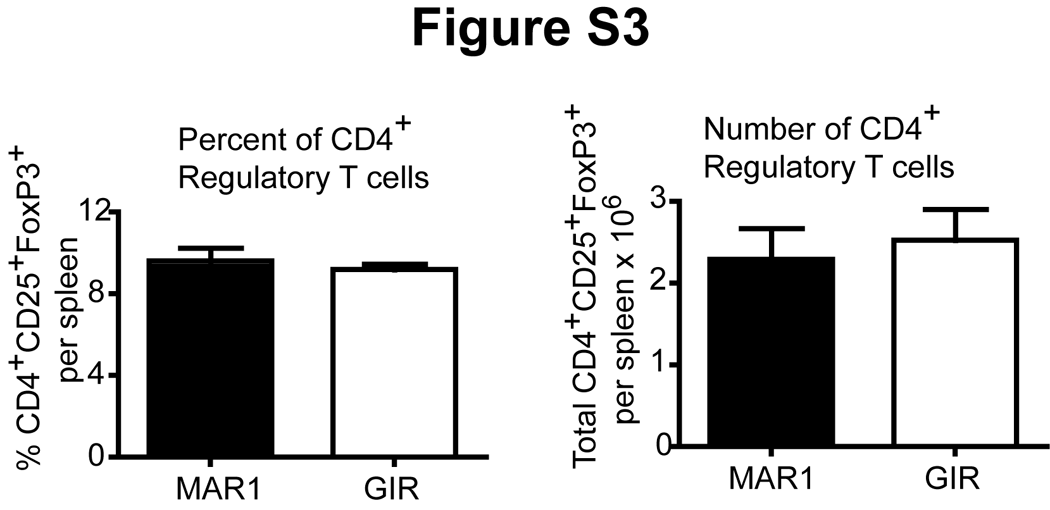

Supplement: Figure S3 — Effect of MAR1-5A3 on Treg development in WNV-infected mice. A. Mice were infected with 102 PFU of WNV and treated with 1 mg of MAR1-5A3 or GIR-208 at four days post infection (n = 8 to 9 mice per group). At day nine after infection, the percentage and number of Tregs from the spleen was determined after staining for CD4, CD25, and FoxP3 and flow cytometric analysis. (TIF) [file ppat.1002407.s003.tif]

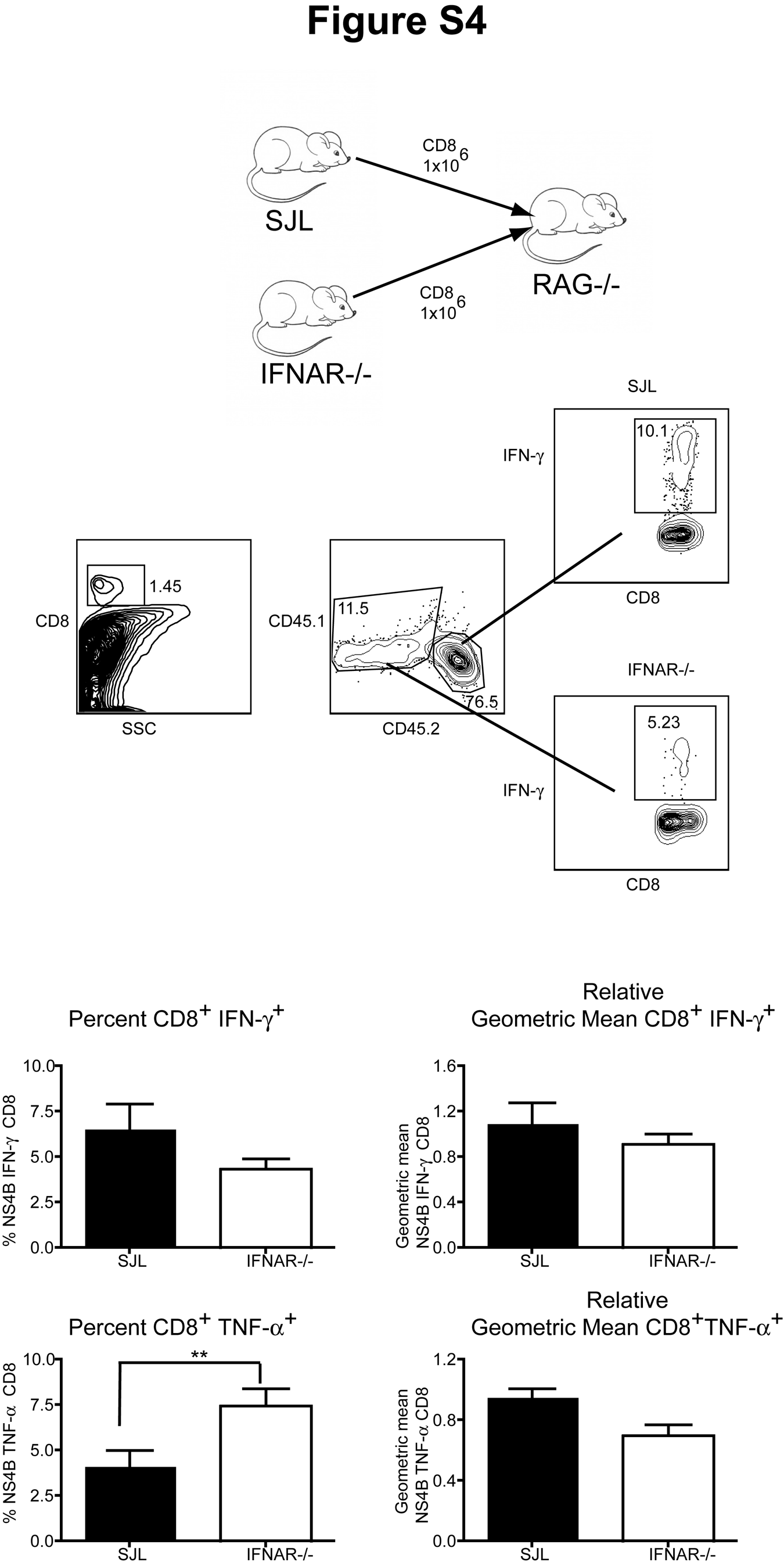

Supplement: Figure S4 — Cell-extrinsic effect of type I IFN signaling in CD8+ T cells on IFN-γ and TNF-α production. An equal number (106 cells) of naïve CD45.2 IFNαβR -/- or CD45.1 B6.SJL purified CD8+ T were adoptively transferred into RAG1 -/- recipient mice (n = 5 mice per group). The following day the mice were infected with 102 PFU of WNV and also phlebotomized to confirm CD8+ T cell transfer (data not shown). At day nine, spleens were harvested and intracellular IFN-γ responses in CD45.1 and C45.2 CD8+ T cells were measured by flow cytometry after ex vivo stimulation with the Db-restricted NS4B peptide. (Top panel) Adoptive transfer strategy. (Middle panels) Gating strategy to distinguish cells from different donors in recipient mice. (Bottom panels) Percentage and relative mean fluorescence intensity of IFN-γ+ and TNF-α+ CD8+ T cells. (TIF) [file ppat.1002407.s004.tif]

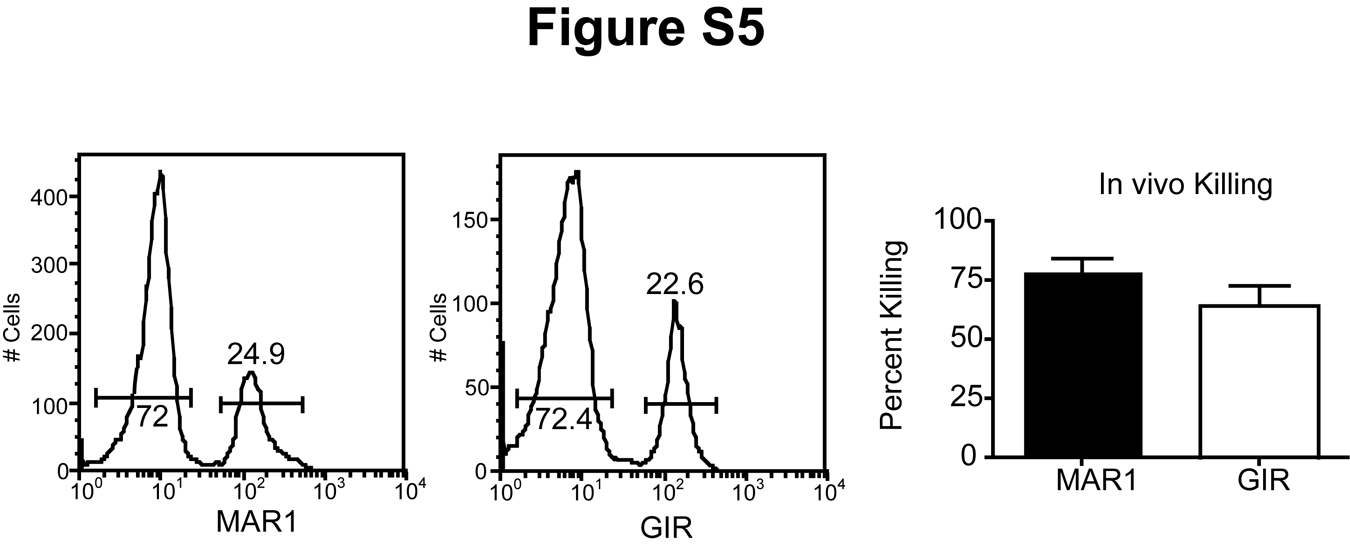

Supplement: Figure S5 — Effect of MAR1-5A3 treatment on CD8+ T cell cytotoxicity in vivo. At day 9 after infection, CFDA-labeled peptide-pulsed CD45.1 naive target cells (splenocytes from B6.SJL mice) were transferred to MAR1-5A3 or GIR-208-treated WNV-infected or naïve mice (n = 6 per group), and six hours later, mice were sacrificed and splenocytes analyzed for the ratio of peptide pulsed to non-pulsed cells by interrogating cells in the CD45.1 gate. The percentage of target cell killing in vivo was calculated by determining the ratio of peptide-pulsed versus unpulsed cells for each mouse, and by normalizing to that seen in naive mice. (TIF) [file ppat.1002407.s005.tif]
